# P2RX7 deficiency prevents tau‐mediated neurodegeneration via regulation of microglial response and suppression of extracellular vesicles containing tau and toxic mitochondrial content

**DOI:** 10.1002/alz70855_103649

**Published:** 2025-12-24

**Authors:** Victor Bodart Santos, Mohammad Abdullah, Justice Ellison, Arun Reddy Ravula, Stephanie Radhakishun, Yang You, Zhewei Liang, Seiko Ikezu, Tsuneya Ikezu

**Affiliations:** ^1^ Mayo Clinic Florida, Jacksonville, FL, USA; ^2^ Mayo Clinic, Jacksonville, FL, USA

## Abstract

**Background:**

We previously identified a novel mechanism describing how pathological tau transfers via extracellular vesicles (EVs) in Alzheimer's disease (AD). Targeting EV secretion to mitigate tau transfer is, therefore, a promising therapeutic approach for AD. P2X purinoreceptor 7 (P2RX7), an ATP‐gated cationic channel predominantly expressed in microglia, regulates the secretion and biogenesis of EVs. Here we investigated the effect of *P2rx7* deficiency in PS19 tauopathy mouse model and by proteomic profiling of brain EVs.

**Method:**

PS19:*P2rx7* ^–/–^ mice at 9‐10 months of age were tested for fear conditioning and their brain sections were assessed for brain atrophy. Tau pathology was evaluated using ELISA and immunofluorescence against phosphorylated (AT8) and misfolded (Alz50) tau. Brain tissues were further assessed by bulk and single cell RNA‐seq (scRNA‐seq) and brain‐EVs were subjected to proteomic profiling by mass‐spectrometry. *P2rx7* ^–/–^ mice were intracranially injected with viral vectors expressing P301L tau in neurons and mEmerald‐CD9 in microglia to visualize the secretion of microglial EVs *in vivo*. Mice conditional knockout (cKO) for *P2rx7* in microglia, neurons and astrocytes were injected with a viral vector expressing P301L tau in entorhinal cortex for tau propagation assessment.

**Result:**

PS19:*P2rx7* ^–/–^ mice showed significant improvement in contextual and cued memory compared to age‐matched PS19 mice, which was accompanied by preserved cortical and hippocampal volume, and significant reduction of hippocampal synapse loss and tau pathology. By weighted gene co‐expression network analysis (WGCNA) we identified a microglial and EV‐related module strongly correlated with tau pathology which was remarkably downregulated in PS19:*P2rx7* ^–/–^ mice hippocampus. PS19 mice showed increased secretion of EVs containing tau and mitochondrial proteins in the mouse brain parenchyma, which was significantly downregulated in PS19:*P2rx7* ^–/–^ mice. scRNA‐seq analysis showed that *P2rx7* is required for microglia conversion to inflammatory phenotype with increased expression of EV genes in PS19 mice. *P2rx7* deficiency reduced microglial mEmerald‐CD9^+^‐EV secretion in mice expressing P301L tau. Moreover, microglia and neuron‐specific deletion of *P2rx7* inhibit tau propagation from the entorhinal cortex to the mouse hippocampus.

**Conclusion:**

Our study demonstrated a microglial P2RX7‐EV axis with potential implications on neuroinflammation and neurodegeneration associated with tau pathology, further indicating the therapeutic potential of targeting *P2rx7* to ameliorate AD progression.

